# Clinical Decision Support for Patient Cases with Asymptomatic Carotid Artery Stenosis Using AI Models and Electronic Medical Records

**DOI:** 10.3390/jcdd12020061

**Published:** 2025-02-06

**Authors:** Mackenzie Madison, Xiao Luo, Jackson Silvey, Robert Brenner, Kartik Gannamaneni, Alan P. Sawchuk

**Affiliations:** 1 Department of Surgery, School of Medicine, Indiana University, Indianapolis, IN 46202, USA; madisonm@iu.edu (M.M.); xiao.luo@okstate.edu (X.L.); robebren@iu.edu (R.B.); kgannama@iu.edu (K.G.); 2 Department of Management Science and Information Systems, Oklahoma State University, Stillwater, OK 74078, USA; jackson.silvey@okstate.edu

**Keywords:** stroke, TIA, carotid stenosis, artificial intelligence, predicting stroke

## Abstract

An artificial intelligence (AI) analysis of electronic medical records (EMRs) was performed to analyze the differences between patients with carotid stenosis who developed symptomatic disease and those who remained asymptomatic. The EMRs of 872 patients who underwent a carotid endarterectomy between 2009 and 2022 were analyzed with AI. This included 408 patients who had carotid intervention for symptomatic carotid disease and 464 patients for asymptomatic, >70% stenosis. By analyzing the EMRs, the Support Vector Machine achieved the highest sensitivity at 0.626 for predicting which of these patients would go on to develop a stroke or TIA. Random Forest had the highest specificity at 0.906. The risk for stroke in patients with carotid stenosis was a balance between optimum medical treatment and the underlying disease processes. Risk factors for developing symptomatic carotid disease included elevated glucose, chronic kidney disease, hyperlipidemia, and current or recent smoking, while protective factors included cardiovascular agents, antihypertensives, and beta blockers. An AI review of EMRs can help determine which patients with carotid stenosis are more likely to develop a stroke to assist with decision making as to whether to proceed with intervention or to demonstrate and encourage reduced stroke risk with risk factor modification.

## 1. Introduction

Intervening to treat patients with asymptomatic carotid stenosis has been controversial in the past and remains controversial today. Original randomized controlled trials comparing the best medical therapy against carotid intervention were performed many years ago and, as all studies do, presented with some inherent flaws. In the interim, the best medical care for atherosclerotic disease and stroke has improved, possibly shifting the risk/benefit ratio of carotid intervention. While the Society of Vascular Surgery presently recommends repairing asymptomatic carotid stenoses of ≥70% in low-surgical-risk patients [1], some vascular interventional luminaries such as Frank Veith, MD, have come to the opinion that they are opposed to intervention for asymptomatic carotid stenosis until good current trials can determine the best candidates [2,3].

In recent years, various machine learning (ML) and artificial intelligence (AI) models have been applied to electronic medical record (EMR) data for disease prediction [4,5,6] and clinical outcome analysis [7,8,9]. These computational models allow for the analysis of vast amounts of chart data to identify patient characteristics that may be linked to specific disease processes. By comparing thousands of variables, ML can uncover differences between patient groups that could indicate a disease association. With the availability of structured data in electronic medical records (EMR), we were able to conduct these analyses efficiently. Our study is the first to use ML and EMR data to identify significant differences in patient characteristics when presenting to a vascular interventionalist with either symptomatic or asymptomatic disease.

## 2. Methods

### 2.1. Data Collection and Processing

This retrospective analysis included the electronic medical record (EMR) data of patients who were categorized as symptomatic vs. asymptomatic according to the criteria of the American Heart Association [10]. Symptomatic carotid artery stenosis includes a stroke, transient cerebral ischemic attack (TIA), or an episode of amaurosis fugax (monocular blindness due to an embolus) in the territory of the carotid artery. Supplemental data included a review of any available CT or MRI brain imaging. The final determination for a symptomatic patient versus an asymptomatic patient (controls) was determined by a physician chart review of the EMR data, including the available CT or MRI brain imaging. In this study, we included 872 patients who had available diagnosis, laboratory tests, and medication data in the EMRs in the Indiana Network for Patient Care (INPC) [11] and who underwent carotid endarterectomy procedures between 2009 and 2022. The additional inclusion criteria were for each patient to have a minimum of one year of EMR data before carotid endarterectomy procedures. There were no exclusion criteria. Based on the chart review criteria, our final cohort included 408 patients who underwent carotid intervention due to symptomatic disease and 464 patients who underwent intervention for asymptomatic stenosis exceeding 70%. This study was approved by the institutional review board (IRB) of Indiana University.

The structured data from the EMR included diagnosis codes (ICD-10), laboratory test results, and medications. For diagnoses, we used the third level of the leaf nodes of the ICD-10 code hierarchy, which groups diagnoses into categories [12]. Pregnancy or childbirth-relevant codes (O00 to O9A) were excluded. For medication, we used the drug group specified in the National Drug Code (NDC) directory as the Established Pharmacologic Class (EPC) [13] and excluded the “medical devices and supplies” and “diagnostic products” categories. Each diagnosis and medication variable was coded as a binary representation (1 or 0), indicating whether the diagnosis was made for the patient or whether medication was prescribed. For laboratory tests, the number values of the laboratory test results were then standardized to “low”, “normal”, or “high” using the laboratory reference range. When multiple results of the same laboratory test were found in the EMR, the most recent result was used. If laboratory tests were not conducted or were absent, they were considered “normal,” operating under the assumption that if any given test result was not available, it was more likely to be normal than abnormal, and we would not be able to conclude otherwise. Each laboratory test result value was coded as 0, 1, or 2, representing “low”, “normal” or “high”.

### 2.2. Machine Learning Algorithms

After applying data processing and normalization steps as described in Section 2.1, the complete study cohort was split into a training set (70% of the complete data) and a test set (30% of the complete data) with a stratified approach. We employed five distinct machine learning models in Python package version 1.3.0, Logistic Regression, Support Vector Machine (SVM), XGBoost, Random Forest, and Multilayer Perceptron, and implemented 10-fold cross-validation on the training dataset to optimize the model parameters. Then, we evaluated and compared the performances of the models on test data. To interpret the factors influencing patients’ likelihood of stroke, TIA, or amaurosis fugax as an indication for intervention compared to those who were asymptomatic, we utilized Shapley Additive exPlanations (SHAP) [14] in Python package version 0.45.0. SHAP has been used when applying ML models for various clinical outcomes or disease prediction [15,16,17]. SHAP is a method used to interpret machine learning models, explaining how each feature contributes to prediction. In the medical domain, SHAP can show which factors (e.g., age, lab results, symptoms) most influence the model’s decision. It assigns each feature with a “SHAP value”, indicating its impact on the outcome. SHAP has different types of explainers for different machine learning models. For example, the Linear Explainer in the SHAP library can be used for linear models, whereas the Tree Explainer in the SHAP library can be used for tree-based models (XGBoost, Random Forest, etc.).

### 2.3. Evaluation Metrics and Statistics Calculations

AUROC (Area Under the ROC Curve), sensitivity, and specificity were used to evaluate the performance of the ML models. AUROC is a metric used to evaluate the performance of a predictive model when distinguishing between two classes (e.g., with vs. without a condition). AUROC measures how well a model distinguishes between patients with and without a condition across all thresholds. The higher the AUROC value, the better the model’s ability to differentiate between positive and negative cases, with a value of 1 indicating perfect discrimination and a value of 0.5 meaning no better than random chance. The *t*-test or chi-squared tests were used to assess the differences in variables between the two groups. The statistical analyses were performed using Python version 3.9.0, and *p*-values < 0.05 were considered statistically significant.

## 3. Results

### 3.1. Statistics of the Study Cohort

The demographic information of the study cohort is summarized as frequency and proportion or as mean and standard deviations, as shown in Table 1. The stroke group was slightly younger and had more female patients. There was no significant difference in the race distribution. The number of encounters per patient was calculated based on all encounters for diagnosis, medications, and lab tests. There was no significant difference in the number of encounters.

### 3.2. Performance Comparison and Interpretation

Table 2 The comparison of the five machine learning models using specificity and sensitivity metrics was done on training and test data, respectively. Random Forest gained the highest specificity (0.906 on test data) and lower sensitivity, whereas SVM shows much higher performance in sensitivity but lower specificity on the test data.

**Figure 1:**
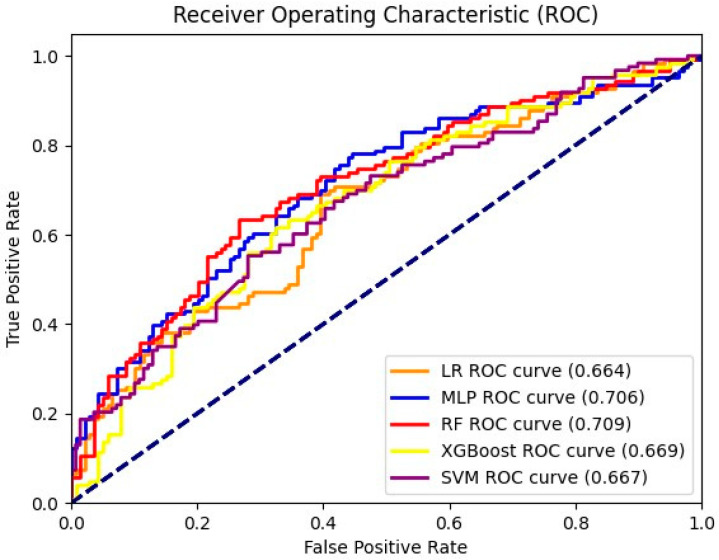
Model comparison based on AUROC curve. The dashed line shows the results of predictions based on random chance.

Figure 1 shows the comparison of the five machine learning models using Area Under the Receiver Operating Characteristics (AUROCs) on the test data. The model with the best performance in terms of the highest AUROC value—0.709—was Random Forest. MLP gained the second-highest AUROC value—0.706—which was slightly lower than Random Forest. The AUROC values of the other three models were much lower.

**Figure 2:**
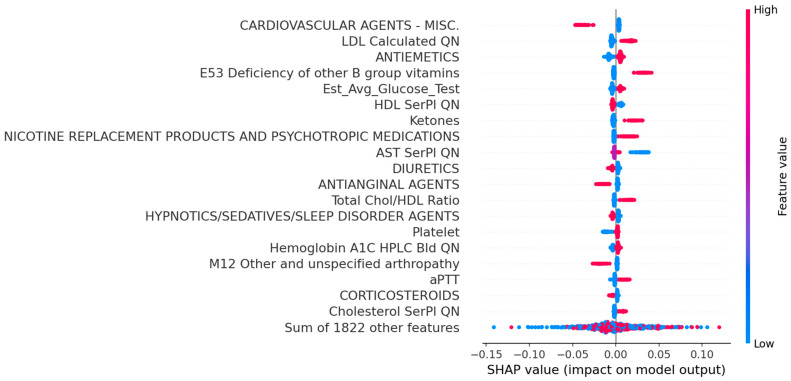
SHAP interpretation of Random Forest. Red indicates a factor making a stroke more likely, while blue indicates a factor making a stroke less likely.

Figure 2 shows the SHAP visualization of the top 20 risk factors identified by the Random Forest model, which includes medications, diagnoses, and laboratory tests. The statistical analysis of these top factors is also included in Table 1. The risk factors for symptomatic carotid disease included elevated glucose, kidney disease, hyperlipidemia, and current smoking status, as evidenced by prescribed nicotine replacement products and psychotropic medications. Protective factors included cardiovascular agents, diuretics, and antianginal agents. Some of the risk factors might only be specific to this study cohort, while the generalizability of these factors to large populations needs further investigation.

### 3.3. Case Study

We further evaluated the proposed method analysis of individual cases to investigate the risk factors that contribute to the development of symptomatic carotid disease. The case studies were analyzed using the local explanation capability of SHAPs visualization. The objective of the local explanations of SHAP is to explain the prediction $f\left(x\right)$ for a given instance x by determining the proportionate contribution of each feature value to a particular outcome. To visualize the local explanation, we used a waterfall plot. The base of the waterfall plot has an expected value of the model output (E[f(x)]), whereas the actual output of the model for each case is given as $f\left(x\right)$ at the top of the plot. If the value of E[f(x)] is larger than $f\left(x\right),$ it is a positive prediction. Otherwise, it is a negative prediction. The waterfall plot organizes features in descending order of importance, with the length of the bars reflecting the proportional feature contribution and red or blue bars reflecting the positive or negative impact of individual features. The top six important features are shown in the waterfall plot for each individual case.

**Figure 3:**
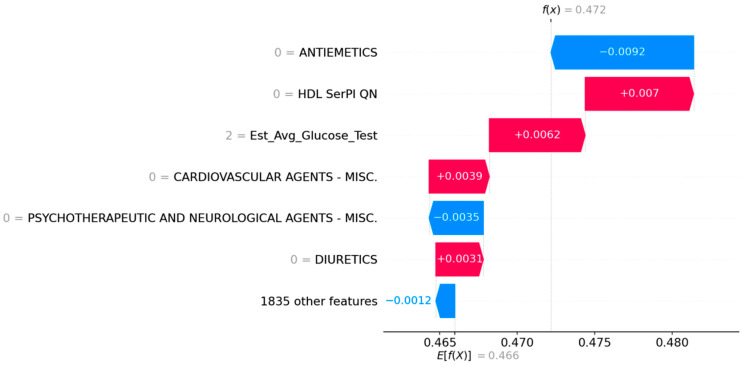
SHAP waterfall plots for Case 1—73-year-old white male. Red factors make a stroke more likely while blue factors make a stroke less likely.

The first case (shown in Figure 3) relates to a 73-year-old white male with symptomatic carotid disease. The waterfall plot shows that low HDL cholesterol levels, high glucose levels, and no medications in the cardiovascular agent group are associated with his symptomatic disease, while the absence of current smoking is a protective factor.

**Figure 4:**
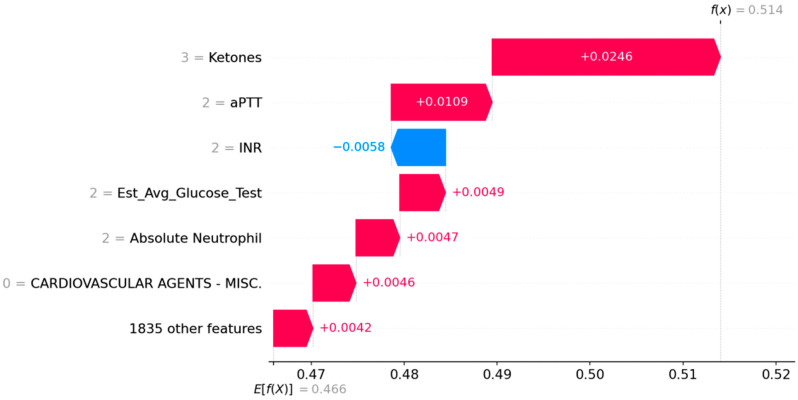
SHAP waterfall plots for Case 2—77-year-old white male. Red factors make a stroke more likely while blue factors make a stroke less likely.

The second case (shown in Figure 4) represents a 77-year-old white male who is symptomatic. High ketones, high activated partial thromboplastin time (aPTT), a high glucose level, high levels of neutrophils, and no medications in the cardiovascular agent group are associated with their symptomatic disease, while a high International Normalized Ratio (INR) is a protective factor.

**Figure 5:**
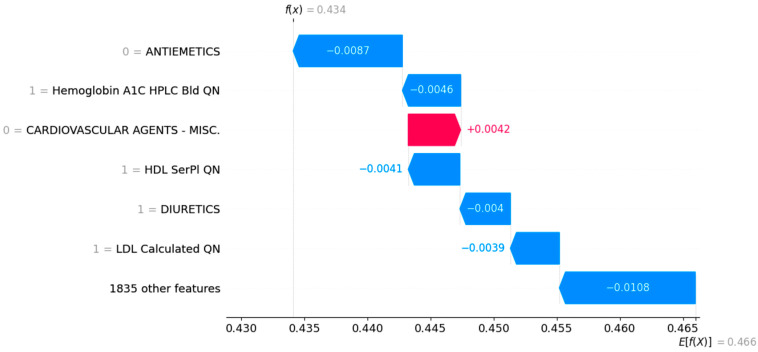
SHAP waterfall plots for Case 3—65-year-old white female. Red factors make a stroke more likely while blue factors make a stroke less likely.

The third case (shown in Figure 5) presents a 65-year-old white female who is an asymptomatic carotid patient. A normal hemoglobin A1C, normal HDL cholesterol level, normal LDL cholesterol level, and no diuretic medications are protective factors, while no medications in the cardiovascular agent group increased the risk of symptomatic disease.

## 4. Discussion

The earliest landmark trials evaluating the risk/benefit ratio of proceeding with a carotid endarterectomy to prevent stroke vs. best medical management were the endarterectomy for asymptomatic carotid stenosis (ACAS) study [18] and the Asymptomatic Carotid Surgery Trial (ACST) study [19]. The ACAS study completed in the 1980s and 1990s projected a 5-year ipsilateral stroke rate of 5.1% for patients undergoing carotid endarterectomy (CEA) for >60% asymptomatic carotid stenosis vs. an 11.0% stroke risk for those patients treated with medical therapy alone. This extrapolated to a 1.2% absolute risk reduction per year in the group undergoing a CEA, but there was an overall 2.3% combined stroke and death rate within 30 days for these patients undergoing surgical intervention. However, about half of the event rates were associated with invasive cerebral angiography, which is seldom performed today and has been replaced by computed tomography angiography (CTA).

The ACST was performed at a later date and published in 2010. At 10 years, the risk for a stroke in patients undergoing a CEA was 10.8% vs. 16.9% for those treated with the best medical therapy for an absolute risk reduction of <1% per year. The stroke risk for medically managed patients decreased in the later years of the study with the increased utilization of statins and antihypertensives.

More recent studies have suggested a decline in the incidence of strokes in patients with carotid stenosis, possibly due to better medical management. In the Oxford Vascular Study, the risk of ipsilateral stroke in patients with ≥50% carotid stenosis was 0.34% per year, while the risk for ipsilateral TIA was 1.78% per year [20]. It should be noted that this study investigated patients with ≥50% stenosis rather than patients with ≥60% stenosis, which may have skewed the results in comparison to previous studies. The Carotid Endarterectomy or Stenting or Best Medical Treatment Alone for moderate-to-severe asymptomatic carotid artery stenosis [21] study that was published in 2022 did not demonstrate a statistically significant difference in the superiority of a CEA or carotid artery stenting (CAS) for stroke prevention compared to best medical management. Using basic Bayesian statistics [22], it can be inferred that the risk of perioperative stroke in patients with asymptomatic carotid artery stenosis could be reduced, and the overall benefit of stroke prevention increased if we could identify the subgroup of patients with asymptomatic carotid stenosis who are most at risk for a stroke due to their medical and target appropriate interventions specifically for them.

Some research has suggested that carotid artery plaque characteristics, as determined by ultrasound, may be useful in determining which cohort of patients with carotid artery plaque are most at risk of becoming symptomatic [23]. However, the United States Preventive Services Task Force has recommended against routine screening for carotid stenosis in asymptomatic patients [23]. A limitation of all of these studies is that the plaque characteristics may significantly change with plaque rupture or ulceration when a patient becomes symptomatic [23,24]. These studies, such as the Carotid Plaque Characteristics Predict Recurrent Ischemic Stroke and TIA [25], rely on evaluating carotid plaque after symptoms have already occurred. However, once a patient develops symptomatic carotid plaque, it is nearly uniformly agreed following the North American Symptomatic Carotid Endarterectomy Trial [26] (NASCET) that those patients are different and carotid intervention should be recommended unless the patient is unable to tolerate the procedure. To truly assess the effectiveness of ultrasound in detecting asymptomatic carotid plaque that could lead to future strokes, one would need to screen asymptomatic patients and monitor them over time. However, this type of study is unlikely to be feasible due to current guidelines that do not support the routine screening of asymptomatic individuals with carotid ultrasound. Additionally, most patients with asymptomatic carotid stenosis tend to remain without symptoms, further limiting the practicality of conducting such a study.

Whether and when to intervene in patients with asymptomatic carotid artery stenosis has been and remains controversial. Most patients with asymptomatic carotid artery stenosis remain asymptomatic, but for those who become symptomatic, a stroke can be devastating or lethal. However, intervention for stroke prevention carries risks of stroke, death, or myocardial infarction. It would be optimal if the vascular interventionalist could separate the cohort of patients who are most at risk of having a future stroke from those at a lesser risk and only intervene in those with a higher risk. One option to separate these cohorts is to analyze their medical records and determine characteristics that place a patient at a higher risk of having a stroke. Simple logic and Bayesian analysis would indicate that separating patients into cohorts of at-risk and least-at-risk would focus surgical care towards patients that would benefit the most from intervention and avoid perioperative risks for those with a lower risk of a stroke.

We tried to compare the best cohorts of patients that we could analyze while having a feasible number of patients in the study, and fairly balanced groups. It would be ideal to have a cohort of patients with asymptomatic carotid artery stenosis who were unable or unwilling to have procedural intervention to determine their natural history. Unfortunately, it is mechanistically and ethically difficult to isolate that cohort. It is acceptable in medical practice to give patients a choice regarding their treatment. Strokes are devastating, and most patients found to have a high-grade carotid artery stenosis choose to have an intervention. There are operative risks for carotid intervention, but they are relatively low since the procedures do not cause major physiological stress. Therefore, there are few patients who would not be candidates for carotid intervention that would be able to be followed to see if they had a stroke without procedural intervention. If we were only evaluating patients with high mortality comorbidities, they might die from other causes before their carotid stenosis became symptomatic. Since only two percent of these asymptomatic patients go on to develop a stroke each year without intervention, it would require following a prohibitively large number of patients with asymptomatic disease to obtain an adequate statistical analysis. If such a study were to be attempted, it would require preliminary clinical data such as those included in this study to support it first.

In this study, we evaluated the performances of various machine learning approaches to analyze structured data in electronic medical records (EMRs) to identify the patients who are most likely to become symptomatic when they have carotid artery stenosis. Based on our knowledge, this study is the first to analyze the EMR data of patients with carotid artery stenosis to estimate the probability of developing symptoms of stroke. The performance comparison demonstrates that traditional machine learning models can deliver solid results, though certain methods may exhibit higher specificity or sensitivity than others. Case studies further reveal that SHAP visualizations facilitate model interpretation, helping to explain how predictions are made. We envision developing a clinical decision support system by integrating multiple machine learning approaches and leveraging the strengths of each model. Implementing these approaches on a large patient cohort would enhance generalizability across various electronic health record (EMR) systems.

Many factors may be associated with asymptomatic vs. symptomatic carotid artery disease. This study evaluated 1842 features listed in medical records. Although clinical judgment is important in medical decision making, it would not be possible for a clinician to analyze the importance of all of these factors for each individual patient. Also, many clinicians assessing patients with carotid artery disease are not specialists in cerebrovascular disease. Large data analysis and artificial intelligence methodology permit the analysis of large quantities of data and make the results available to all clinicians.

Many of the factors associated with the development of atherosclerotic plaque were also found to be associated with symptomatic atherosclerotic embolic disease after the plaque had formed [26]. An initial examination of some of the associated factors was somewhat bewildering. Our initial evaluation using specified codes showed that the use of psychotherapeutic and neurologic agents is associated with a higher risk for symptomatic disease, which was not initially an expected finding. Delving into that category of medications revealed that it included nicotine patches, nicotine gum, varenicline, and bupropion, all of which are used for smoking cessation [27]. Further consideration indicates that this category is a proxy for current cigarette smoking. Hyperhomocystinemia has been associated with the development of atherosclerotic plaque [28]. Vitamin B deficiencies, specifically vitamin B12 and folate, may elevate homocysteine levels. This may be the reason that vitamin B deficiencies are found to be associated with symptomatic carotid artery disease. Some of the relationships found require further investigation.

Ideally, it would be more beneficial to have better sensitivities, specificities, and areas under the receiver operator curve (AUROC) than the values obtained in this study. However, at present, the clinical world is generally divided between clinicians who recommend carotid procedural intervention in all patients with an acceptable risk who have greater than approximately 70% stenosis and those clinicians who recommend that procedures should never be performed in patients with asymptomatic carotid stenosis because of the low stroke risk of two percent per year. At present, a clinician recommending against carotid intervention will only be wrong two percent of the time, but if that recommendation is incorrect, the resulting stroke may devastate the patient. This paper hopes to identify a common clinical approach by finding a cohort of patients with greater than 70% carotid arterial stenosis who might receive more benefit from a carotid intervention.

This study identified the characteristics associated with patients most at risk of having symptomatic carotid artery disease in a large group of patients presenting to vascular surgeons for evaluation. The risk factors found may not be directly causative but may indicate that the patient has an underlying disease process that is being picked up indirectly in the EMR. Until further analysis is performed, a medication associated with symptomatic carotid artery disease may have a relationship to the findings because of the underlying disease process it is treating or because of the effects of the medication itself. This study does have some limitations in that it did not analyze patients with asymptomatic disease who did not present to a vascular surgeon for evaluation; this was also a single-center study. Confirmation of these findings will require evaluating a larger and more diverse cohort of patients. It could also potentially be enhanced by assessing carotid duplex ultrasound data, although plaque characteristics may vastly change after a patient becomes symptomatic. Obtaining carotid duplex data from patients before they become symptomatic would be required and is seldom performed in clinical practice unless a patient is found on a screening test to have significant carotid artery disease. Future studies will try to include additional medical centers and available duplex carotid ultrasound data.

The authors envision that this data analysis could be automatically integrated into the EMR. Patients with carotid stenosis with a high risk of developing symptomatic disease could be identified before they have a stroke, which may be devastating or lethal. An appropriate vascular physician could then discuss these findings with the patient. Options, including proceeding with a carotid stent, endarterectomy, or risk factor modification, could be given. Risk factor modification only works if the risk factors are modified. If the patient does not think that they can adhere to specific aspects of risk factor modification, such as smoking cessation or the improved control of underlying risk factors, or if these changes are not adequately implemented by the patient in a set period of time, the placement of a carotid stent or proceeding with a carotid endarterectomy should be considered after quantifying the patient’s operative risks.

## 5. Limitations of the Study

Our research aimed to evaluate the performance of machine learning models in identifying patients who are asymptomatic for carotid stenosis and who may later develop stroke symptoms using electronic medical record (EMR) data. We acknowledge several limitations to this study. The cohort size was relatively small, though it did include patients with diverse comorbidities, necessitating further validation to assess the generalizability of our approach to larger populations. Additionally, this study focused solely on structured data; incorporating natural language processing techniques, such as large language models [29,30], could help extract valuable clinical features from unstructured EMR data, potentially improving model performance. Clinical imaging data, which may offer predictive insights through plaque characteristics [31], was not used in this study but could be explored in future research. Lastly, we recognize the need for human experts to validate the relevance of clinical features in each case. We also foresee the importance of human-in-the-loop AI approaches to continually refine and validate AI outputs in future studies.

## 6. Conclusions

This study demonstrates that an in-depth machine learning-based approach to analyzing the medical record may be used to sort out patients with carotid disease who are more likely to develop a stroke or symptomatic carotid artery disease from those who may be more likely to remain asymptomatic in order to direct intervention with its inherent risks to those patients who might benefit the most or to encourage risk factor modification in order to decrease stroke risk. It demonstrates the relationship between disease processes, which increase the risk of having a carotid embolic stroke, and the treatment of these risk factors, lessening the risk of stroke in patients who have developed carotid artery stenosis. Together with the additional analysis of duplex ultrasound and computed tomography to evaluate plaque characteristics, it may help physicians limit carotid intervention to the cohort of patients that would benefit from it the most.

## Figures and Tables

**Table 1:** Study cohort summary and analysis.

	Total	Asymptomatic	Symptomatic	*p*-Value
Number of Patients	872	464	408	
Encounters per Patient, mean (STD)	24.24 (21.00)	25.20 (19.79)	23.14 (22.21)	0.151
**Demographics**				
Age, mean (std)	68.99 (10.51)	69.89 (10.29)	67.98 (10.69)	0.007
Gender				<0.05
Male	423	293 (63.1%)	230 (56.4%)	
Female	349	171 (36.9%)	178 (43.6%)	
Race				0.315
White	793	430 (92.7%)	363 (90.0%)	
African American	65	27 (5.8%)	38 (9.3%)	
Asian	7	3 (0.6%)	4 (1.0%)	
Other or Unknown	7	4 (0.9%)	3 (0.7%)	
**Medication**				
Cardiovascular Agents	87	71 (15.3%)	16 (3.9%)	<0.01
Antiemetics	449	273 (58.8%)	276 (67.6%)	<0.01
Psychotherapeutic and Neurological Agents	124	45 (9.7%)	79 (19.4%)	<0.01
Diuretics	359	211 (45.5%)	148 (36.3%)	<0.01
Antianginal Agents	97	62 (13.3%)	35 (8.6%)	<0.05
Hypnotics/Sedatives/Sleep Disorder Agents	404	243 (52.4%)	161 (39.5%)	<0.01
Corticosteroids	229	135 (29.1%)	94 (23.0%)	0.051
**Laboratory Test**				
LDL Calculated QN (high)	182	67 (14.4%)	115 (28.2%)	<0.01
Est Avg Glucose Test (high)	348	151 (32.5%)	197 (48.3%)	<0.01
HDL SerPl QN (low)	566	329 (70.9%)	237 (58.1%)	<0.01
Ketones (high)	63	24 (5.2%)	39 (9.6%)	<0.05
AST SerPl QN	111	49 (10.3%)	63 (15.4%)	<0.05
AST SerPl QN (low)	59	21 (4.5%)	38 (9.3%)	
AST SerPl QN (high)	52	27 (5.8%)	25 (6.1%)	
Total Chol/HDL Ratio (high)	98	33 (7.1%)	65 (15.9%)	<0.01
Platelet	129	78 (16.8%)	51 (12.5%)	0.100
Platelet (low)	112	70 (15.1%)	42 (10.3%)	
Platelet (high)	17	8 (1.7%)	9 (2.2%)	
Hemoglobin A1C Bld QN	505	240	265	<0.01
Hemoglobin A1C Bld QN (low)	4	0 (0%)	4 (0.1%)	
Hemoglobin A1C Bld QN (high)	501	240 (51.7%)	261 (64.0%)	
aPTT	100	40 (8.6%)	60 (14.7%)	<0.05
aPTT (low)	6	3 (0.6%)	3 (0.7%)	
aPTT (high)	94	37 (8.0%)	57 (14.0%)	
Cholesterol SerPl QN (high)	114	42 (9.1%)	72 (17.6%)	<0.01
**Diagnosis**				
E53 Deficiency of other B Group Vitamins	88	26 (5.6%)	52 (12.7%)	<0.01
M12 Other and Unspecified Arthropathy	62	41 (8.8%)	21 (5.1%)	<0.05

**Table 2:** Performance comparison using sensitivity and specificity.

Models	Sensitivity		Specificity	
	Training	Test	Training	Test
LR	0.856	0.569	0.901	0.626
SVM	**0.852**	**0.626**	0.923	0.626
MLP	0.684	0.569	0.818	0.726
RF	0.533	0.317	**0.978**	**0.906**
XGBoost	0.715	0.455	0.870	0.776

Note: The bold numbers produced the best performances in terms of sensitivity or specificity.

## Data Availability

The original contributions presented in this study are included in the article. Further inquiries can be directed to the corresponding author.
